# Prognostic Role of Left Ventricular Systolic Function Measured by Speckle Tracking Echocardiography in Septic Shock

**DOI:** 10.1155/2020/7927353

**Published:** 2020-10-21

**Authors:** Pham Dang Hai, Nguyen Thanh Binh, Nguyen Viet Quang Hien, Nguyen Huy Hoang, Vu Ngoc Hoan, Pham Nguyen Son, Le Thi Viet Hoa

**Affiliations:** ^1^Intensive Care Unit, 108 Military Central Hospital, Vietnam; ^2^Department of Anesthesiology and Critical Care Medicine, Hue Central Hospital, Vietnam; ^3^Department of Diagnostic Imaging, 108 Military Central Hospital, Vietnam; ^4^Military Medical University, Vietnam; ^5^Department of Cardiology, 108 Military Central Hospital, Vietnam; ^6^Intensive Care Unit, Tam Anh General Hospital, Vietnam

## Abstract

**Background:**

Left ventricular (LV) systolic dysfunction is common in septic shock. Global longitudinal strain (GLS) measured by speckle tracking echocardiography (STE) is a useful marker of intrinsic left ventricular systolic function. However, the association between left ventricular GLS and outcome in septic patients is not well understood. We performed this prospective study to investigate the prognostic value of LV systolic function utilizing speckle tracking echocardiography in patients with septic shock.

**Methods:**

All the patients with septic shock based on sepsis-3 definition admitted to the intensive care unit were prospectively studied with STE within 24 hours after the onset of septic shock. Baseline clinical and echocardiographic variables were collected. The primary outcome was in-hospital mortality.

**Results:**

During a 19-month period, 90 consecutive patients were enrolled in the study. The in-hospital mortality rate was 43.3%. Compared with survivors, nonsurvivors exhibited significantly less negative GLS (−13.1 ± 3.3% versus −15.8 ± 2.9%; *p* < 0.001), which reflected worse LV systolic function. The area under the ROC curves of GLS for the prediction of mortality was 0.76 (95% CI 0.67 to 0.87). Patients with GLS > −14.1% showed a significantly higher mortality rate (67.7% versus 15.6%; *p* < 0.0001; log‐rank = 23.3; *p* < 0.0001). In the multivariate analysis, GLS (HR, 1.27; 95% CI 1.07 to 1.50, *p* = 0.005) and SOFA scores (HR, 1.27; 95% CI 1.08 to 1.50, *p* = 0.004) were independent predictors of in-hospital mortality.

**Conclusions:**

Our study indicated that LV systolic function measured by STE might be associated with mortality in patients with septic shock.

## 1. Introduction

Septic shock is a leading cause of intensive care unit mortality and is often associated with multiorgan dysfunction [1]. Although there are recent advances in earlier recognition and management, the mortality rate in septic shock remains high (>40%) [2]. Multiorgan dysfunction is seen in more than 45% of patients with septic shock and is associated with worse prognosis [3, 4]. Cardiovascular dysfunction in septic shock may manifest as circulatory failure, myocardial dysfunction, or myocardial injury. Sepsis-induced myocardial dysfunction can include left ventricular (LV) systolic dysfunction or LV diastolic dysfunction or right ventricular dysfunction and related to a significantly increased mortality rate of 70–90% [5, 6]. However, the diagnosis of septic cardiomyopathy remains challenging [7].

Two-dimensional echocardiography is a noninvasive, low-cost imaging technique in evaluating cardiac function in sepsis and septic shock [8]. LV ejection fraction (LVEF) obtained from conventional echocardiography is most commonly used to access LV systolic function. However, it has several limitations, such as depending on fluid status, afterload, and poor prognostic value in patients with septic shock [9–12].

Speckle tracking echocardiography is emerging as a better tool of intrinsic LV function. LV global longitudinal strain (GLS) measured by speckle tracking echocardiography (STE) is a more reliable, reproducible, and sensitive modality for evaluating LV systolic function [13–15]. GLS is introduced as a predictive marker of cardiovascular events and mortality [8, 14]. Although several studies have shown the association of LV GLS with outcome in patients with septic shock, evidence of the prognostic value of LV GLS in these patients remains a limitation [16–18].

Therefore, the aim of this study was to evaluate the prognostic value of a LV systolic function using speckle tracking echocardiography in patients with septic shock. We hypothesized that worse LV GLS (less negative values, representing LV systolic dysfunction) is associated with increased mortality in patients with septic shock.

## 2. Materials and Methods

### 2.1. Study Population

The observational, prospective cross-sectional study was performed in a twenty-five-bed intensive care unit (ICU) of 108 Military Central Hospital, Vietnam. Between May 2017 and December 2018, all patients aged 18 years or older admitted for septic shock that developed within 24 hours before ICU admission were screened for eligibility. Septic shock was defined according to the sepsis-3 definition with the criteria of sepsis, combined with persisting hypotension requiring vasopressors to keep a mean arterial pressure ≥ 65 mmHg and a serum lactate level greater than 2 mmol/L despite adequate fluid resuscitation [2]. The patients were treated according to the guidelines of the Surviving Sepsis Campaign 2016 [4].

Exclusion criteria included the presence of ischemic heart disease, heart failure, moderate to severe valvular disease, valve replacement surgery, cardiac arrhythmia, postcardiac arrest, insufficient echocardiographic image quality, and patients or their relatives declining participation.

Baseline clinical variables including age, gender, comorbidities, hemodynamic parameters, vasopressor dose, SOFA score [19], and APACHE II score [20] were obtained and calculated within 24 h of ICU admission. Echocardiographic parameters were collected within 24 h of diagnosis of septic shock. All patients were followed up till the hospital discharge, and the primary endpoint was all-cause in-hospital mortality.

The study was approved by the institutional ethical committee of 108 Military Central Hospital. All participating patients or their legal representatives provided written informed consent.

### 2.2. Echocardiography and Two-Dimensional Speckle Tracking

Two-dimensional conventional echocardiography and speckle tracking echocardiography were performed using a commercially available ultrasound system (Vivid S5, GE Healthcare, USA). All echocardiograms were performed by cardiologists with advanced training in echocardiography.

Echocardiographic cine loops were obtained by recording a minimum of three consecutive cardiac cycles. Images were obtained at a frame rate of 50–90 frames/s and digitally transferred to dedicated software for offline analysis.

LV end-diastolic volume (LVEDV), LV end-systolic volume (LVESV), LV end-diastolic diameters (LVEDD), LV end-systolic diameters (LVESD), LV fractional shortening, and LV ejection fraction (LVEF) based on modified biplane Simpson's method in the apical four- and two-chamber views were measured according to the American Society of Echocardiography guidelines [21].

Speckle tracking echocardiography analysis was performed for each patient using offline software with the EchoPAC workstation (version 112, GE Healthcare, USA). GLS was calculated by means of two-dimensional speckle tracking strain from the three standard apical views (three-chamber, two-chamber, and four-chamber views). The software automatically traced a region of interest, including the entire myocardium, by using a point-and-click approach. The myocardial tracking was verified, and the region-of-interest width was manually adjusted to achieve optimal alignment if needed. Thereafter, the region of interest of apical images outlining the entire left ventricular wall was divided into 6 standard segments, and six corresponding time-strain curves were generated. GLS was determined to average the peak systolic values of the 18 segments, derived from the 6 segments of 3 standard apical views (two- and four-chamber and apical long-axis views) [21]. GLS is expressed as a percent change (%). Negative values of GLS represent the ability of myocardial contractility. All analysis was measured by two independent observers to estimate interobserver variability.

### 2.3. Statistical Analysis

Data analysis was performed using SPSS version 20.0 software (SPSS, Inc., Chicago, IL, USA). Continuous variables were expressed as mean values and standard deviation. Categorical variables were presented as frequencies and percentages. Comparisons were performed using Student's *t*-tests or Mann-Whitney test for continuous variables. Categorical variables were compared with the Chi-squared test or a Fisher exact test, as appropriate.

A receiver operating characteristic (ROC) curve analysis was performed to determine the cut-off value of GLS for the prediction of in-hospital mortality. The best cut-off point was defined by choosing the maximum value of Youden's index, which was calculated as “sensitivity + specificity − 1” [22]. Survival analysis was conducted using the Kaplan-Meier method, and survival rates were compared according to the cut-off value of GLS by the log-rank test.

Univariate Cox regression analyses were performed to identify the factors related to in-hospital mortality. Variables with *p* values < 0.05 were integrated into the multivariate regression model by using means of stepwise selection. There was an absence of multicollinearity between variables in the multivariate model. Finally, 20 patients were randomly selected to test the interobserver and intraobserver variability for GLS measurements by using Bland-Altman analysis. *p* values < 0.05 were considered statistically significant.

## 3. Results

### 3.1. Patient Characteristics

During the study period, from May 2017 to December 2018, a total of 124 consecutive patients with septic shock were admitted to the ICU. 34 patients were excluded from analysis due to moderate to severe valvular disease (*n* = 5), prior cardiac surgery (*n* = 2), ischemic heart disease (*n* = 4), death before performing echocardiography (*n* = 4), postcardiac arrest (*n* = 2), and poor image echocardiography quality (*n* = 17). Thus, 90 patients were analyzed.

The mean age was 68.8 ± 15.1 years, 67 patients were male (74.4%), SOFA score is 10.7, and APACHE II score is 20.1. The prevalence of comorbidities includes the following: hypertension (46%), diabetes (28%), chronic kidney failure (21.1%), stroke (13.3%), and COPD (2.2%). The sources of infection were the respiratory tract (47.8%), abdomen (38.9%), urinary tract (5.6%), soft tissue (6.6%), and unknown origin (1.1%). The median duration of ICU and hospitalization was 7.9 days and 19.1 days, respectively. The in-hospital mortality rate was 43.3% (*n* = 39).

Table 1 compares baseline clinical variables between the nonsurvival group and the survival group. There were no significant differences in age, gender, comorbidities, or source of infection. The proportion of mechanical ventilation, SOFA score, and APACHE II score were significantly higher in the nonsurvival group compared with the survival group (*p* < 0.05).

Table 2 summarizes the hemodynamic and echocardiographic parameters on day 1. The heart rate, CVP, cardiac output, and the dose of norepinephrine were not significantly different between the two groups. Mean arterial pressure under norepinephrine was significantly lower in nonsurvivors (67.9 ± 9.9 versus 74.6 ± 12.1 mmHg; *p* = 0.006).

### 3.2. Echocardiographic Variables

There were no significant differences in FS and LVEF between the nonsurvivors and the survivors (Table 2). However, the nonsurvivors had less negative GLS than the survivors, indicating a worse LV systolic function in the nonsurvivors.

### 3.3. ROC Curve Analysis

**Figure 1:**
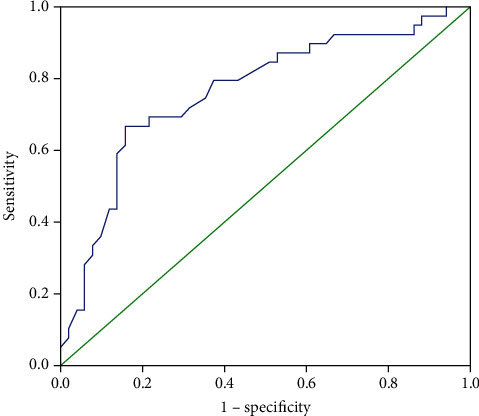
Receiver operating characteristic (ROC) curve for predicting in-hospital mortality by using the left ventricular global longitudinal strain (GLS). Area under the curve is 0.76 (cut-off: −14.1%, Se: 67%, Sp: 74%).

**Figure 2:**
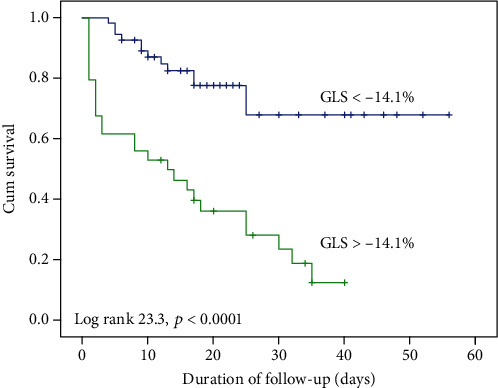
The in-hospital mortality in the study population classified according to the left ventricular global longitudinal strain (GLS) < −14.1% or GLS > −14.1%.

The results of ROC analysis for GLS in predicting in-hospital mortality are showed in Figure 1; the area under the curve is 0.76. The optimal cut-off GLS value is −14.1%; the sensitivity and specificity to predict in-hospital mortality were 67% and 74%, respectively. Patients with GLS > −14.1% had a significantly higher mortality rate (67.7% versus 15.6%; *p* < 0.0001; log‐rank = 23.3; *p* < 0.0001) (Table 2 and Figure 2).

### 3.4. Logistic Regression Analysis

Univariate analysis using the logistic Cox regression model showed that the SOFA score and GLS were associated with the risk of in-hospital mortality (Table 3). In the multivariate analysis, GLS remained the independent predictor of in-hospital mortality in septic shock patients (HR, 1.27; 95% CI 1.07 to 1.50; *p* = 0.005). Moreover, the SOFA score was also an independent predictor.

### 3.5. Reproducibility of GLS Measurements

The intraclass correlation coefficient (ICC) for interobserver concordance was 0.93 (95% CI 0.83 to 0.97) for GLS. ICC intraobserver concordance was 0.95 (0.87-0.98). Bland-Altman analysis showed good intra- and interobserver agreement with a small nonsignificant bias for GLS.

## 4. Discussion

The main finding of our study showed that LV systolic dysfunction based on GLS is associated with mortality, and reduced (less negative) GLS is an independent predictor of in-hospital mortality in patients with septic shock.

Myocardial dysfunction, referred to as septic cardiomyopathy, is one of the common findings in septic shock. Dalla et al. [23] and Post et al. [24] have demonstrated that the LV systolic dysfunction (LVSD) in septic shock was 50% to 60%. Several factors contribute to the subclinical deterioration of LV systolic function, such as toxins, microvascular vasoconstriction in the subendocardial muscle layer [25], myocardial depressant factor, proinflammatory mediators, and mitochondrial dysfunction [6]. More and more studies showed a correlation between LVSD and mortality [10, 11, 17].

Global longitudinal strain measured by STE might be considered a good surrogate of intrinsic LV systolic function contrary to LVEF due to dependence on LV loading conditions, especially preload changes [26].

Our results are similar to previous studies in which authors evaluated the predictive value of GLS in septic shock. Chang et al. reported that septic shock patients based on sepsis-2 with reduced LV systolic function by GLS had higher ICU and in-hospital mortality [11]. GLS was an independent predictor of ICU and in-hospital mortality [11]. Similar to the findings of Ricarte-Bratti et al. [27], the LV systolic function measured by STE was significantly lower in nonsurvivors. Palmieri et al. [17] found a worse LV GLS in nonsurvivors at 7 days and 28 days (-9.1% vs. -10.8%; *p* < 0.05). A meta-analysis by Sanfilippo et al. [16] showed that worse GLS value is associated with higher mortality in patients with severe sepsis or septic shock.

However, several studies showed the contrary results. Orde et al. [28] failed to find any difference in LV systolic function between survivors and nonsurvivors. Similar to the study of Geer et al. [18], they did not find evidence of LV systolic dysfunction in survivors. Landesberg et al. (2014) did not demonstrate GLS to be an independent predictor of mortality in patients with severe sepsis and septic shock (odds ratio: 1.16; *p* = 0.06). These might be explained due to the small sample size (*n* = 50 to 60) [18, 28], a heterogeneous population, and difference in diagnostic criteria of septic shock [28].

The optimal GLS cut-off point of predicting in-hospital mortality in septic shock remains uncertain. In our study, the receiver operating characteristics (ROC) for parameter GLS showed fair discrimination (area under the curve (AUC) 0.76) for predicting in-hospital mortality with an optimal cut-off of −14.1%. These findings were in agreement with those of Chang et al. [11]; GLS of -13% had the best sensitivity and specificity in predicting mortality in patients with septic shock (sensitivity 76%, specificity 82%, and AUC 0.79). Hassanin et al. [29] studied 32 patients with sepsis and found that the area under the curve of GLS to predict mortality was 0.9 (95% CI 0.32 to 0.48), with optimal cut-off value at −16.8% (sensitivity 100%, specificity 86%). The intrinsic differences between populations, along with proprietary software and vendors of STE technology, could contribute to the observed differences.

In the multivariate analysis, GLS and the SOFA score were the independent outcome predictors in septic shock patients. Similar to Chang et al. [11], less negative GLS and the APACHE II score were independent predictors of ICU and hospital mortalities.

Our results support the hypothesis that LV systolic dysfunction measured by STE at baseline could be useful for the prognosis of patients with septic shock based on the sepsis-3 definition.

## 5. Limitations

Our study had several limitations. Firstly, the design of this study was single-center and the small sample size. Thus, our results lack external validation. Larger, prospective, multicenter studies regarding the predictive value of STE in septic shock could be considered in the future. Secondly, repeated imaging and further STE analysis were not performed during the course of hospitalization, so reversibility of LV systolic dysfunction could not be assessed. Moreover, a longitudinal echocardiographic study may provide additional information regarding the clinical progression of sepsis. Thirdly, patients with septic shock admitted to the ICU had different underlying diseases and severity, so it was difficult to assess the cause of death accurately. Fourthly, speckle tracking echocardiography is a new imaging technique requiring adequate image quality, which could be a challenge, especially in patients with mechanical ventilation [30]. Finally, other speckle tracking parameters to evaluate LV systolic function, such as global circumferential and radial strain, which is showed to be promising, were not obtained [30].

## 6. Conclusion

Our study has shown that LV systolic function measured by speckle tracking echocardiography via GLS might be associated with mortality in patients with septic shock. However, further studies involving more patients are needed to validate the results of this study.

## Figures and Tables

**Table 1:** Baseline characteristics and comparison between survivors and nonsurvivors at the onset of septic shock (day 1).

	Survivors (*n* = 51)	Nonsurvivors (*n* = 39)	*p* value
Characteristics			
Age, mean (years)	69.1 ± 13.1	68.4 ± 17.4	0.843
Male (%)	67 (74.4)	33 (89.2)	0.987
SOFA score	9.5 ± 2.5	12.2 ± 3.6	<0.001^∗^
APACHE II score	16.9 ± 7.0	24.1 ± 7.2	<0.001^∗^
Mechanical ventilation, *n* (%)	42 (82.3)	38 (97.4)	0.024
CRRT, *n* (%)	25 (49)	26 (68.4)	0.068
ICU LOS (days)	7.9 ± 6.8	7.9 ± 7.6	0.997
Hospital LOS (days)	23.7 ± 12.7	11 ± 9.8	<0.001^∗^
Comorbidities
Hypertension, *n* (%)	21 (41.2)	16 (41.0)	0.989
Diabetes mellitus, *n* (%)	10 (19.6)	12 (30.8)	0.222
Stroke, *n* (%)	7 (13.7)	5 (12.8)	0.900
CKD, *n* (%)	10 (16.9)	9 (23.1)	0.689
COPD, *n* (%)	1 (2.0)	1 (2.6)	0.847
Liver disease, *n* (%)	11 (21.6)	5 (12.8)	0.282
Source of infection			0.027
Abdominal	20 (39.2)	23 (59.0)	
Respiratory	26 (51.0)	9 (23.1)	
Kidney	3 (5.9)	2 (5.1)	
Skin	1 (2.0)	5 (12.8)	
Other	1 (2.0)	0 (0.0)	
Bacteremia	15 (29.4)	17 (43.6)	0.164
Gram-positive	2 (13.4)	2 (12.6)	
Gram-negative	11 (73.0)	12 (68.6)	
Others	2 (13.4)	3 (18.8)	

Data are presented as means ± SD and number (*n*) of patients (%), as appropriate. APACHE II: acute physiology and chronic health evaluation; COPD: chronic obstructive pulmonary disease; CKD: chronic kidney disease; CRRT: continuous renal replacement therapy; ICU: intensive care unit; LOS: length of stay; SOFA: sequential organ failure assessment. ^∗^*p* < 0.05.

**Table 2:** Baseline hemodynamic and echocardiographic data of survivors and nonsurvivors at the onset of septic shock (day 1).

	Survivors (*n* = 51)	Nonsurvivors (*n* = 39)	*p* value
Hemodynamic parameters			
HR (beat/min)	99.0 ± 17.3	106.8 ± 19.7	0.233
Mean blood pressure (mmHg)	74.6 ± 12.1	67.9 ± 9.9	0.006^∗^
CVP (mmHg)	7.5 ± 2.4	6.9 ± 3.0	0.296
Norepinephrine (*μ*g/kg/min)	0.32 ± 0.30	0.47 ± 0.53	0.218
Conventional echocardiography			
LVEDV (mL)	101.2 ± 36.7	96.5 ± 32.3	0.529
LVESV (mL)	37.0 ± 17.0	36.0 ± 16.1	0.771
LVEDD (mm)	46.1 ± 7.1	45.1 ± 6.9	0.530
LVESD (mm)	30.0 ± 5.4	29.6 ± 5.6	0.764
LVEF (%)	59.1 ± 9.6	56.9 ± 10.3	0.317
FS (%)	34.9 ± 5.6	34.5 ± 6.2	0.751
LVOT	20.2 ± 1.7	19.8 ± 1.7	0.261
VTI (mm)	19.9 ± 3.5	17.8 ± 4.3	0.018
CO (L/min)	6.3 ± 1.6	6.1 ± 2.1	0.619
Speckle tracking echocardiography			
LS-A4C (%)	−15.7 ± 2.9	−12.8 ± 3.1	<0.001^∗^
LS-A2C (%)	−15.5 ± 3.4	−12.8 ± 3.9	0.001^∗^
LS-A3C (%)	−16.3 ± 3.3	−13.7 ± 3.7	0.001^∗^
GLS (%)	−5.8 ± 2.9	−13.1 ± 3.3	<0.001^∗^
GLS > −14.1%	8 (15.6%)	26 (66.7%)	<0.001^∗^

Data are presented as means ± SD. A3C: apical 3-chamber view; A4C: apical 4-chamber view; A2C: apical 2-chamber view; CO: cardiac output; CVP: central venous pressure; GLS: global longitudinal strain by speckle tracking echocardiography; FS: fractional shortening; LS: longitudinal strain; LVEDV: left ventricular end-diastolic volume; LVESV: left ventricular end-systolic volume; LVESD: left ventricular end-systolic dimension; LVEDD: left ventricular end-diastolic dimension; LVEF: left ventricular ejection fraction; LOVT: left ventricular outflow tract; HR: heart rate; VTI: velocity-time integral. ^∗^*p* < 0.05.

**Table 3:** Univariable and multivariate analyses for predictors of in-hospital mortality in patients with septic shock.

	Univariable		Multivariable	
Dependent variables	Hazard ratio (95% CI)	*p*	Hazard ratio (95% CI)	*p*
Age	0.99 (0.97-1.02)	0.84	—	—
Male gender	0.99 (0.38–2.58)	0.98	—	—
Hypertension	0.99 (0.42–2.32)	0.99	—	—
Diabetes mellitus	1.82 (0.69–4.80)	0.22	—	—
CKD	1.23 (0.44–3.40)	0.69	—	—
Noradrenalin dose	2.43 (0.78–7.57)	0.12	—	—
SOFA score	1.34 (1.14–1.57)	<0.001	1.27 (1.08–1.50)	0.004
GLS	1.34 (1.14–1.59)	<0.001	1.27 (1.07–1.50)	0.005
Reduced GLS (GLS > −14.1%)	10.75 (3.93–29.5)	<0.001		

CI: confidence interval; CKD: chronic kidney disease; GLS: global longitudinal strain; SOFA: sequential organ failure assessment.

## Data Availability

The data used to support the findings of this study are available from the corresponding authors upon request.

## Abbreviations

APACHE: Acute physiology and chronic health evaluation
A3C: Apical 3 chamber view
A4C: Apical 4 chamber view
A2C: Apical 2 chamber view
AUC: Area under the curve
COPD: Chronic obstructive pulmonary disease
CI: Confidence interval
CKD: Chronic kidney disease
CRRT: Continuous renal replacement therapy
CVP: Central venous pressure
EF: Ejection fraction
FS: Fractional shortening
GLS: Global longitudinal strain
HR: Heart rate
ICC: Intraclass correlation coefficient
ICU: Intensive care unit
LS: Longitudinal strain
LV: Left ventricular
LVEDV: Left ventricular end-diastolic volume
LVESV: Left ventricular end-systolic volume
LVESD: Left ventricular end-systolic dimension
LVEDD: Left ventricular end-diastolic dimension
LOVT: Left ventricular outflow tract
SOFA: Sequential organ failure assessment
STE: Speckle tracking echocardiography
TDI: Tissue Doppler imaging
VTI: Velocity-time integral.
